# Multifocal Gastrointestinal Melanoma Metastases More Than Two Decades After Primary Diagnosis: Complete Resection and Long‐Term Follow‐Up

**DOI:** 10.1002/ccr3.73903

**Published:** 2026-11-10

**Authors:** Per Nyman, Marcus Gustavsson, Olof Söderman, Roger Olofson Bagge, Jenny Nyqvist‐Streng

**Affiliations:** ^1^ Department of Surgery Skaraborg Hospital Skövde Region Västra Götaland Sweden; ^2^ Department of Clinical Pathology Skaraborg Hospital Skövde Region Västra Götaland Sweden; ^3^ Department of Clinical Radiology Skaraborg Hospital Skövde Region Västra Götaland Sweden; ^4^ Department of Surgery, Institute of Clinical Sciences, Sahlgrenska Academy University of Gothenburg Gothenburg Sweden

**Keywords:** case report, cutaneous melanoma, gastrointestinal metastases, late metastatic presentation, metastasectomy

## Abstract

Melanoma may present with gastrointestinal metastases decades after primary diagnosis. A man treated for cutaneous melanoma in 1992 had local recurrence in 1993 and an in‐transit metastasis in 2013. Intestinal metastases were resected in 2017. No further recurrence was documented through June 2026. Gastrointestinal symptoms or unexplained anemia warrant investigation.

## Key Clinical Message

New gastrointestinal symptoms or unexplained anemia in patients with a history of melanoma warrant investigation for metastases, even decades after primary diagnosis. Multifocal intestinal involvement should prompt multidisciplinary assessment of disease extent and resectability rather than automatic exclusion from consideration of complete surgical resection.

## 1 Introduction

Cutaneous melanoma is an aggressive malignancy characterized by a marked propensity for metastatic spread and considerable biological heterogeneity. Although melanoma accounts for a minority of skin cancer diagnoses, it is responsible for the vast majority of skin cancer‐related deaths due to its metastatic potential [1]. One of the most remarkable features of melanoma is its capacity for prolonged tumor dormancy. Disseminated melanoma cells may persist in a clinically silent state for years or even decades before reactivation and metastatic progression become apparent [2]. While most melanoma recurrences occur within the first 2–5 years after diagnosis, late recurrence remains a well‐recognized phenomenon. In a large institutional series including 4731 melanoma patients, approximately 6.9% of patients experienced recurrence ten or more years after initial treatment [3]. Extremely late recurrence, defined as recurrence occurring more than 20 years after primary diagnosis, is considerably rarer and has been reported predominantly in isolated case reports and small case series. The mechanisms underlying late clinical presentations remain incompletely understood [3].

The gastrointestinal tract represents one of the most frequent sites of melanoma dissemination. Autopsy studies have demonstrated gastrointestinal involvement in approximately 50%–60% of patients dying from disseminated melanoma [4]. In contrast, gastrointestinal metastases are diagnosed clinically in only 1%–5% of melanoma patients during life, highlighting the substantial discrepancy between clinical recognition and true disease prevalence [5, 6]. Among gastrointestinal sites, the small intestine represents the most frequent location for metastatic involvement, accounting for the majority of clinically detected gastrointestinal melanoma metastases [6].

We report four gastrointestinal metastases diagnosed more than two decades after primary cutaneous melanoma and approximately 3 years after an in‐transit metastasis. This late presentation after prior recurrence cannot establish an uninterrupted disease‐free interval or the duration of any biologic dormancy.

## 2 Case Presentation

A married elderly male former smoker with no documented alcohol misuse was followed over several decades for melanoma originating from the medial aspect of the right lower leg. Past medical history was significant for Type 2 diabetes mellitus treated with oral hypoglycemic agents, aortic stenosis, aortic sclerosis, and hyperlipidemia. No family history of malignancy was documented.

In 1992, he underwent a diagnostic excision of a pigmented lesion from the right lower extremity where histopathological examination demonstrated a superficial spreading melanoma with Breslow thickness of 1.0 mm and Clark Level III. Wide local excision was subsequently performed with free margins. One year later, in 1993, a local recurrence developed which was treated with repeat wide excision with a 2 cm margin. Histopathological examination confirmed recurrent melanoma, and tumor‐free surgical margins were achieved.

The patient remained clinically disease‐free for approximately 20 years. In April 2013, a 4‐cm in‐transit melanoma metastasis was detected medial to the right knee and completely excised with clear margins. Sentinel lymph node biopsy from the right inguinal basin showed no metastatic involvement. Histopathological examination confirmed metastatic melanoma, and molecular analysis showed no BRAF mutation. Serum S‐100 was mildly elevated at 0.13 μg/L (reference < 0.11 μg/L). At a regional melanoma conference on May 28, 2013, no additional treatment was recommended.

## 3 Methods (Differential Diagnosis, Investigations and Treatment)

Three years later, in December 2016, the patient developed progressive fatigue and altered bowel habits. Laboratory investigations demonstrated severe anemia and CT of the chest and abdomen demonstrated multifocal intestinal disease without reported widespread visceral dissemination (Figure 1A–D). A preoperative brain CT performed with and without intravenous contrast on January 5, 2017 showed no intracranial metastases. Preoperative colonoscopy confirmed an approximately 3‐cm polypoid tumor in the descending colon (Figure 2). Exploratory laparotomy was performed in February 2017. Intraoperative examination revealed four distinct intestinal metastatic lesions. Two separate ulcerated metastatic deposits were identified in the jejunum. A third metastatic lesion was found in the ileum. A fourth lesion was located in the left colon. All four lesions were macroscopically distinct and non‐contiguous. Segmental resections of the involved jejunal and ileal segments were performed with primary anastomoses, together with resection of the affected colonic segment.

**FIGURE 1:**
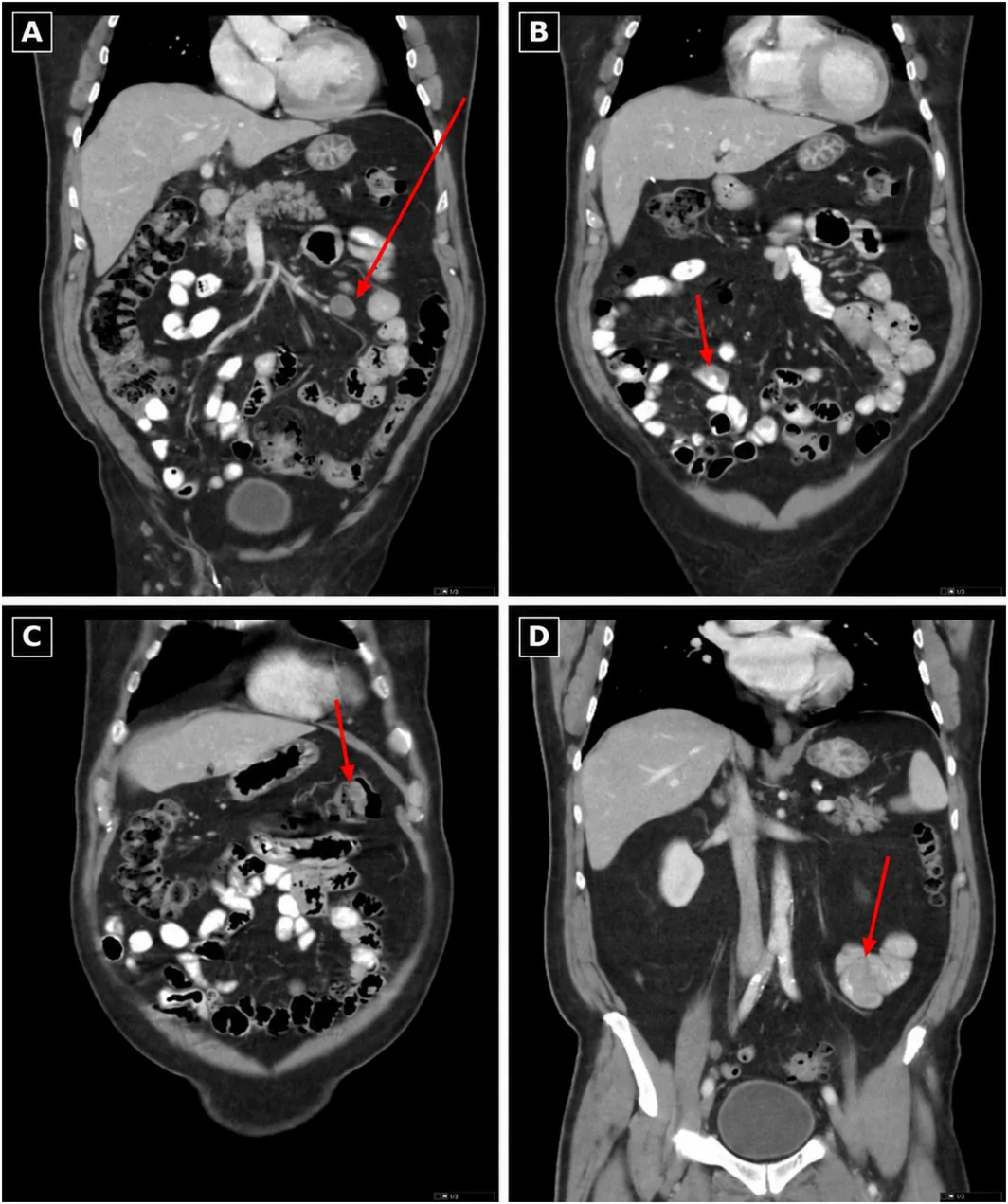
Contrast‐enhanced computed tomography of the abdomen, coronal reformations (5‐mm slice thickness; soft‐tissue window). (A) The arrow indicates an approximately 3‐cm polypoid tumor in the left descending colon. (B–C) Coronal images at different anteroposterior levels demonstrate the additional arrow‐marked intra‐abdominal findings in relation to adjacent bowel loops and mesentery. (D) A more posterior coronal image demonstrates the arrow‐marked finding adjacent to the left kidney. The panels illustrate the overall multifocal intra‐abdominal disease; a one‐to‐one correspondence between each arrow‐marked CT finding and each of the four subsequently resected lesions was not documented.

**FIGURE 2:**
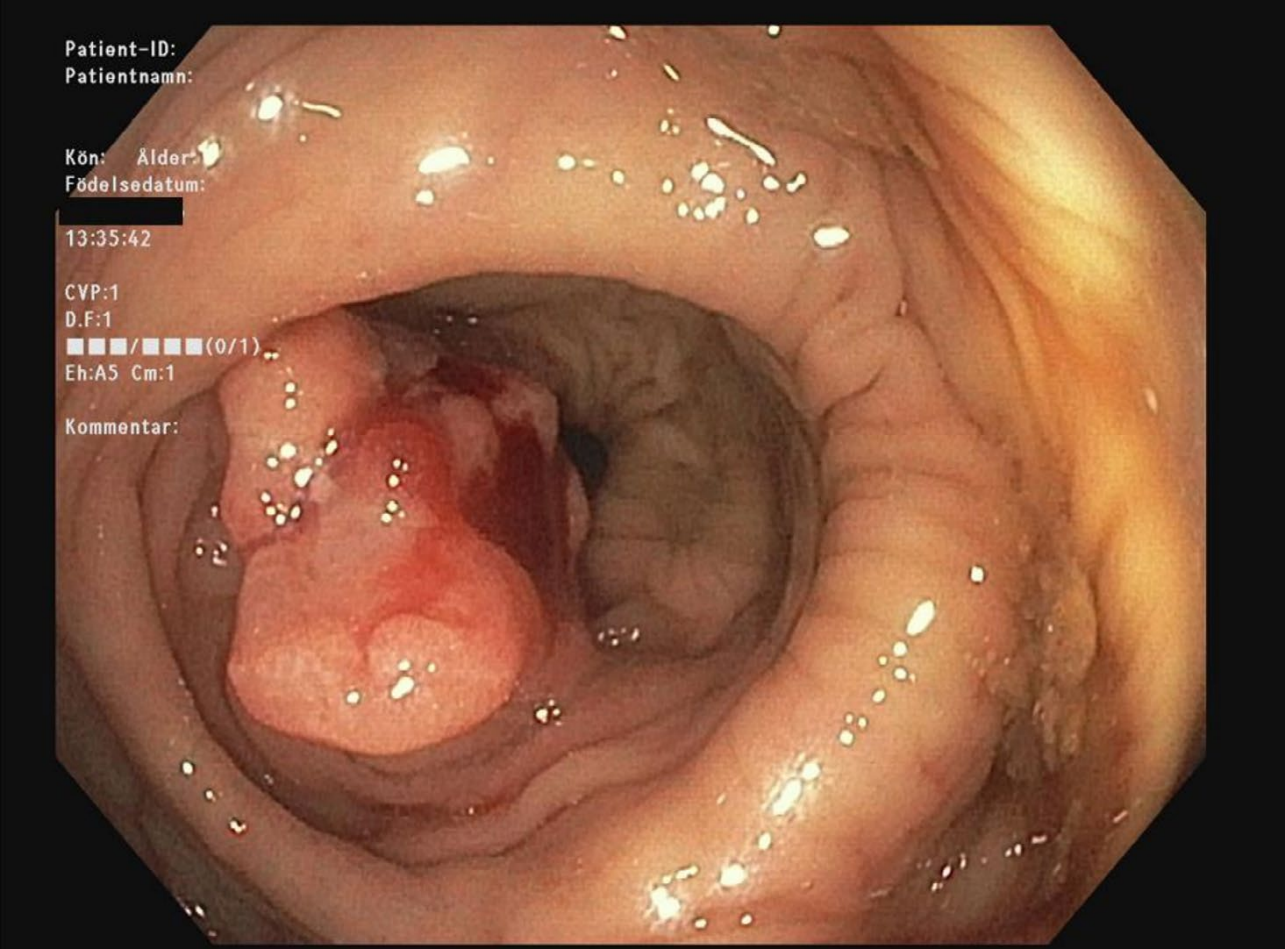
Colonoscopy demonstrates an approximately 3‐cm polypoid tumor in the descending colon.

Histopathological examination confirmed multifocal metastatic malignant melanoma in all four lesions. The two jejunal metastases infiltrated the mucosa and muscularis propria without serosal penetration. The ileal lesion demonstrated similar morphology with transmural invasion limited to the bowel wall. The colonic lesion involved the bowel wall with histological features consistent with metastatic melanoma rather than primary colorectal malignancy (Figure 3A–F). The illustrated colonic lesion showed nuclear SOX10 positivity and no AE1/AE3 or CDX2 expression (Figure 3D–F). Resection margins were negative in all specimens, and no metastatic involvement was identified in the examined mesenteric lymph nodes.

**FIGURE 3:**
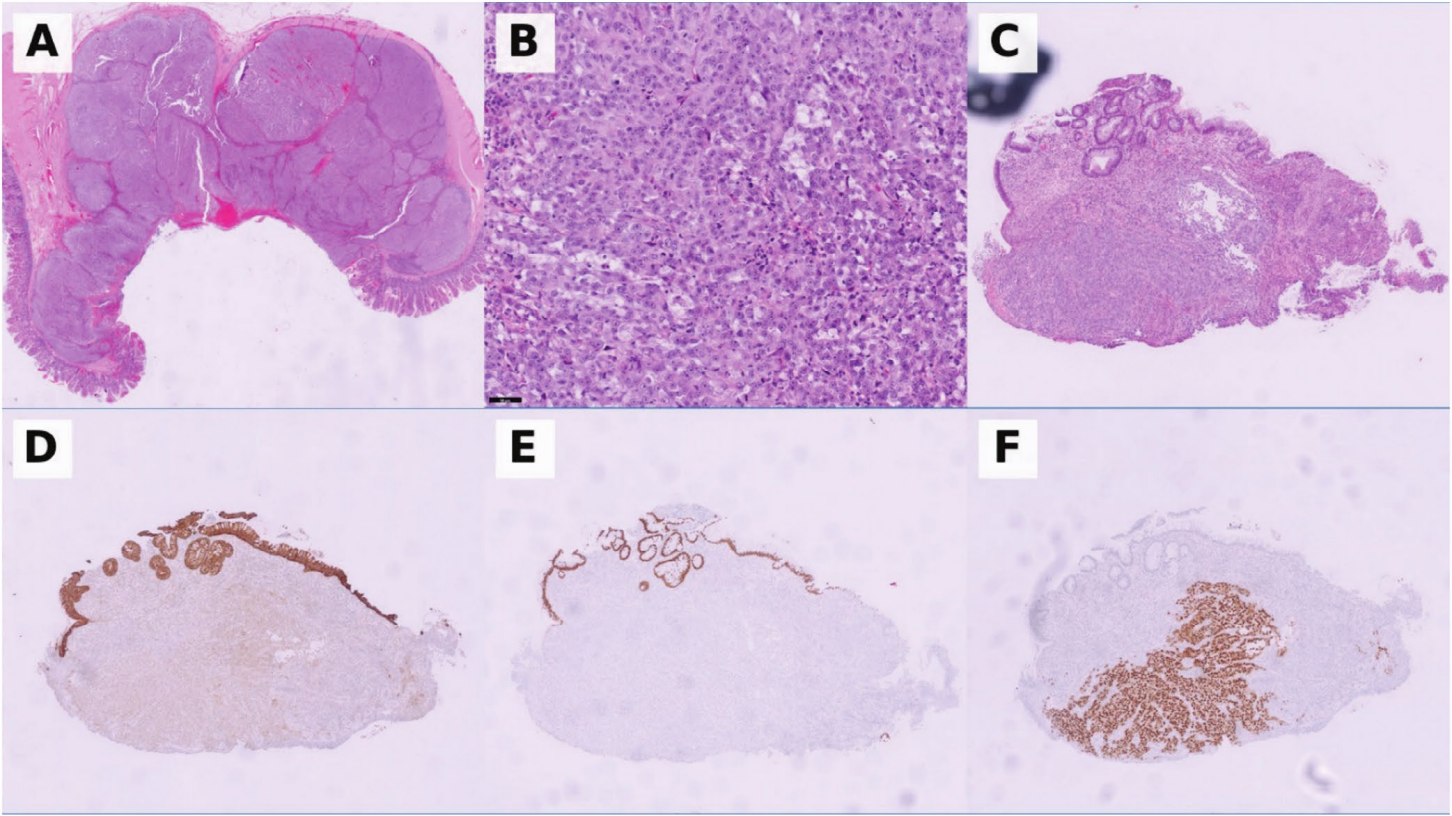
(A) Histopathological overview of the colonic lesion from the resection specimen demonstrating infiltration of the bowel wall by a malignant neoplasm (H&E, 2×). (B) Sheets and nests of malignant tumor cells (H&E, 20×). (C) Preoperative biopsies show infiltration of the same neoplasm (H&E, 5×). Tumor cells are (D) negative for AE1/AE3, arguing against epithelial differentiation (5×), (E) negative for CD×2, arguing against intestinal epithelial differentiation (5×), (F) with a strong nuclear SO×10 expression supporting melanocytic differentiation (5×).

## 4 Outcome and Follow‐Up

The postoperative course was uncomplicated. The patient recovered well, with normalization of bowel function and gradual correction of anemia. A postoperative follow‐up brain CT performed with and without intravenous contrast on June 10, 2017 showed no intracranial metastases. At a regional melanoma conference on June 13, 2017, LD and S‐100 were described as within the reference range and no BRAF mutation had been identified. No additional adjuvant treatment was recommended. Monthly laboratory monitoring was planned, with reassessment for systemic immunotherapy if the marker values increased. During an evaluation for suspected stroke in December 2021, brain CT on 6 and 8 December and brain MRI on 9 December showed neither stroke nor brain metastases. No further recurrence or metastatic disease was documented through June 2026 (Table 1).

**TABLE 1:** Clinical timeline and serial laboratory values.

Timepoint	Clinical event	S‐100 (μg/L)	LD (μkat/L)	Hb (g/L)	Creatinine (μmol/L)
1992	Primary cutaneous melanoma of right lower leg diagnosed; local excision performed	—	—	—	—
1993	Local recurrence; wide re‐excision with negative margins	—	—	—	—
2013‐05‐08	In‐transit metastasis near the right knee excised; inguinal sentinel node negative	**0.13 ↑**	—	151	90
2013‐05‐28	Regional melanoma conference: no additional treatment recommended	—	—	—	—
2016‐12‐12	Presentation with gastrointestinal symptoms and anemia; colon lesion identified	—	—	**73 ↓**	108
2016‐12‐20	Colonoscopic biopsy confirms metastatic melanoma to colon	0.06	1.8	115	109
2017‐01‐05	Brain CT with and without IV contrast: no brain metastases	—	—	—	—
2017‐02‐14	Surgical resection of multifocal intestinal metastases (jejunum, ileum, left colon)	—	—	—	—
2017‐05‐04	Early postoperative oncologic surveillance	**0.15 ↑**	2.4	105	104
2017‐06‐10	Brain CT with and without IV contrast: no brain metastases	—	—	—	—
2017‐06‐13	Regional melanoma conference: no adjuvant treatment; monthly laboratory follow‐up; reassess systemic immunotherapy if markers rise	—	—	—	—
2017‐07‐17	Postoperative normalization	0.04	1.7	110	79
2017‐10‐16	Continued surveillance	0.07	1.6	138	108
2017‐12‐27	Stable follow‐up	0.04	1.4	141	106
2018‐07‐10	Routine follow‐up	0.10	1.6	141	—
2018‐10‐03	Stable disease surveillance	0.05	1.8	150	91
2019‐01‐07	Ongoing surveillance	0.06	2.0	149	—
2019‐07‐08	Surveillance laboratory assessment	0.04	1.7	136	—
2021‐10‐21	Long‐term surveillance	—	—	158	112
2021‐12‐08	Brain CT on 8 Dec and MRI on 9 Dec for suspected stroke: neither stroke nor brain metastases	—	—	—	—
2022‐01‐03	Stable follow‐up	0.06	1.7	—	—
2023‐02‐10	Continued biochemical stability	0.063	1.9	151	122
2024‐03‐24	Surveillance	0.076	1.6	—	—
2025‐04‐15	Surveillance	0.059	1.9	—	—
2025‐06‐10	Mild anemia noted	—	—	**124 ↓**	—
2025‐10‐31	Follow‐up recovery	—	—	146	118
2026‐05‐13	Latest surveillance	0.059	1.8	—	—

*Note:* A dash indicates that no result was available in the reviewed record; ↑ and ↓ denote values flagged as high and low, respectively.

## 5 Discussion

The gastrointestinal metastases were diagnosed approximately 24 years after the primary cutaneous melanoma and approximately 3 years after resection of an in‐transit metastasis. Because the patient did not have an uninterrupted 24‐year recurrence‐free interval, the clinical course cannot establish when dissemination occurred or whether melanoma cells remained dormant before the gastrointestinal metastases became apparent. Comparing the intestinal lesions with the original melanoma and the 2013 metastasis, preferably using morphological and molecular methods, could have strengthened the assessment of their relationship. However, the earlier specimens are no longer available for comparison. Although clinical history and the intestinal pathology support metastatic melanoma, a shared clonal origin cannot be demonstrated definitively, and an occult second primary melanoma cannot be excluded with certainty.

This case underscores the rarity of clinically detected multifocal gastrointestinal melanoma metastases. Although gastrointestinal involvement has been demonstrated in up to 60% of patients at post‐mortem examination, symptomatic gastrointestinal metastases are clinically diagnosed in only 1%–5% of melanoma patients during life [6, 7]. Furthermore, the presence of four synchronous and anatomically distinct intestinal metastases involving jejunum, ileum, and colon represents an exceptionally uncommon pattern of disease dissemination.

Perhaps the most important clinical lesson from this case relates to the role of surgery. Historically, gastrointestinal melanoma metastases were associated with advanced disease and poor survival; outcomes have since improved with modern systemic therapies and, in selected patients, complete resection [6]. However, retrospective studies have reported favorable outcomes after complete resection in selected patients with limited gastrointestinal metastatic disease [8, 9, 10, 11]. More recently, contemporary series from high‐volume melanoma centers have demonstrated that favorable outcomes after metastasectomy remain achievable also in the era of immune checkpoint inhibitors, particularly in patients who respond to systemic therapy and can be rendered free of macroscopic disease following surgery [12, 13]. The role of metastasectomy has consequently evolved from a stand‐alone treatment modality to an integrated component of multimodal melanoma care. Current concepts distinguish surgery performed with the intention of achieving no evidence of disease (NED), resection of oligometastatic disease, resection of isolated or oligoprogressive lesions while maintaining otherwise effective systemic therapy (non‐progressive residual disease, NPRD), and palliative surgery for symptom control. Careful patient selection, multidisciplinary decision‐making, and assessment of response to systemic therapy are increasingly recognized as key determinants of outcome [14].

In this patient, complete macroscopic resection of four separate intestinal lesions was feasible, and no further recurrence was documented during more than 8 years of follow‐up. This favorable course cannot establish the effect of surgery in a single case. It illustrates the importance of assessing disease extent and resectability in multidisciplinary discussion, even when more than one intestinal lesion is present.

In the era of effective systemic therapies, the role of metastasectomy depends on the clinical context and should be considered alongside other available treatment options rather than inferred from an individual outcome.

## 6 Conclusion

Multifocal gastrointestinal melanoma metastases may present more than two decades after primary diagnosis, including after intervening recurrence. In this patient, complete resection of four intestinal lesions was followed by prolonged follow‐up without documented recurrence. This single case cannot establish a treatment effect but supports multidisciplinary assessment of selected patients with potentially resectable disease. New gastrointestinal symptoms or unexplained anemia in a patient with a melanoma history warrant investigation.

## Author Contributions

**Per Nyman:** conceptualization, methodology, project administration, validation, investigation, writing – original draft, resources, writing – review and editing. **Marcus Gustavsson:** investigation, validation, visualization, writing – original draft, formal analysis, writing – review and editing. **Olof Söderman:** investigation, validation, writing – original draft, visualization, formal analysis. **Roger Olofson Bagge:** writing – original draft, validation, writing – review and editing. **Jenny Nyqvist‐Streng:** conceptualization, investigation, writing – original draft, writing – review and editing, methodology, validation, visualization, supervision, project administration, resources.

## Funding

The authors have nothing to report.

## Consent

Oral and written informed consent for publication was obtained from the patient and is available for review by the Editor‐in‐Chief upon request.

## Conflicts of Interest

The authors declare no conflicts of interest.

## Data Availability

The data that support the findings of this study are available from the corresponding author upon reasonable request.
